# Myeloid-Derived Suppressor Cells: Major Figures that Shape the Immunosuppressive and Angiogenic Network in Cancer

**DOI:** 10.3390/cells8121647

**Published:** 2019-12-15

**Authors:** Eleni-Kyriaki Vetsika, Aristeidis Koukos, Athanasios Kotsakis

**Affiliations:** 1 Department of Medicine, pMEDgr, School of Health Sciences, National and Kapodistrian University of Athens, 11527 Athens, Greece; ekvetsika@med.uoa.gr; 2 Laboratory of Translational Oncology, School of Medicine, University of Crete, P.O. Box 2208, 71003 Heraklion, Greece; aris.tidisk@hotmail.com; 3 Department of Medicine, School of Health Sciences, University of Thessaly, 41334 Larissa, Greece; 4 Department of Medical Oncology, University General Hospital of Larissa, 41334 Larissa, Greece

**Keywords:** myeloid-derived suppressor cells, immunosuppression, angiogenesis, cancer immunology, tumor microenvironment, vascular endothelial growth factor receptor

## Abstract

Myeloid-derived suppressor cells (MDSCs) constitute a vast population of immature myeloid cells implicated in various conditions. Most notably, their role in cancer is of great complexity. They exert immunosuppressive functions like hampering cancer immunity mediated by T lymphocytes and natural killer cells, while simultaneously they can recruit T regulatory cells to further promote immunosuppression, thus shielding tumor cells against the immune defenses. In addition, they were shown to support tumor invasion and metastasis by inducing vascularization. Yet again, in order to exert their angiogenic activities, they do have at their disposal a variety of occasionally overlapping mechanisms, mainly driven by VEGF/JAK/STAT signaling. In this concept, they have risen to be a rather attractive target for therapies, including depletion or maturation, so as to overcome cancer immunity and suppress angiogenic activity. Even though, many studies have been conducted to better understand these cells, there is much to be done yet. This article hopes to shed some light on the paradoxal complexity of these cells, while elucidating some of the key features of MDSCs in relation to immunosuppression and, most importantly, to the vascularization processes, along with current therapeutic options in cancer, in relation to MDSC depletion.

## 1. Introduction

Until recently, myeloid-derived suppressor cells (MDSCs) composed a taboo in the field of cancer immunology, since it is a vast and heterogeneous population of immature cells of the immune system [1,2,3,4]. These cells derive from hematopoietic stem cells (HSCs) residing in bone marrow (BM), which give rise to the immature myeloid cell (IMC) population [2]. Normally, under the right combination of growth factors, the IMC population gives rise to all of the terminally differentiated myeloid cells such as neutrophils, macrophages, and dendritic cells (DCs) [2]. However, a malfunction in the maturation process of this ancestral population favors the maintenance of a pool of MDSCs [5]. MDSCs can arise under different circumstances in cancer. When there is need for more myeloid cells, a program called emergency myelopoiesis is activated in the BM, giving rise to MDSCs from the IMC population [6,7]. In the periphery, a similar procedure is initiated, called extramedullary myelopoiesis [8]. The precursor cells, due to tumor-derived factors, might migrate out of the bone marrow into the blood, peripheral tissue, and lymph nodes. These cells would then proliferate and become MDSCs through activation at extramedullary sites [9]. A novel hypothesis also suggests that MDSCs may arise as a part of reprogramming of the existing differentiated myeloid cells (monocytes and polymorphonuclear cells) [9,10,11]. In any case, the development of MDSCs is governed by multiple signals found in their microenvironment (e.g., colony stimulating factors, growth mediators, and cytokines) that retain the ability of these cells to survive and stay undifferentiated [9]. Once the MDSC population is established in the immune system, it is then free to execute its numerous functions, e.g., cancer progression [5].

Given the fact that the MDSC population is actually comprised of a bounty of different cells, it is difficult to determine their actual phenotype. Nonetheless, it is evident that there are two distinct subpopulations within the major MDSC population. To begin with, a monocytic population (M-MDSC) is distinguished in mice by the expression of the surface markers CD11b and Ly6C, along with a polymorphonuclear subpopulation (PMN-MDSC) characterized by means of CD11b and Ly6G [2]. As far as the characterization of the equivalent population in humans is concerned, the exact combination of markers still poses a challenge [12,13]. Regardless, some phenotypes were proposed for both the M-MDSC and the PMN-MDSC subpopulations. M-MDSCs were established as CD14^+^CD15^−^CD11b^+^CD33^+^HLA-DR^−^Lin^−^, as well as CD14^+^CD15^+^CD11b^+^CD33^+^HLA-DR^−^Lin^−^, whereas the PMN-MDSC subpopulation was designated as CD14^−^CD15^+^CD11b^+^CD33^+^HLA-DR^−^Lin^−^ or CD11b^+^CD14^−^CD66b^+^ [13,14,15]. Recently, another MDSC subtype was proposed, called early-stage MDSC (eMDSC), which lucks the markers for both monocytic and granulocytic populations, baring the phenotype of Lin^−^HLA-DR^−^CD33^+^CD11b^+^CD14^−^CD15^−^ [13,15,16,17,18,19]. These cell populations not only exist as free cells in the peripheral blood, but also as enriched cell populations in the tumor microenvironment (TME) [20]. In the latter, MDSCs acquire a far more suppressive ability, with the M-MDSC population and the classical activated monocytes (M1) rapidly evolving into tumor-associated macrophages (TAMs), while the neutrophils tend to transform in a more suppressive subpopulation, the tumor-associated neutrophils (TANs) [1,15,21].

Despite this generic discrimination between the two MDSC populations, a bias still exists regarding the accuracy of their nomenclature. This issue arises during the characterization of tumor-infiltrating myeloid (TIM) cells [22]. Apart from MDSCs, other myeloid cells like macrophages (M1 and M2/TAMs), neutrophils (and TANs), and DCs reside in the tumor tissue [22,23]. Some of these cells share a common phenotypes like PMN-MDSCs and neutrophils, or even functions [13], thus complicating the phenotypic characterization. To overcome this crucible, researches have been focused on using single-cell transcriptomics to characterize myeloid populations. Zilionis et al. demonstrated that the myeloid landscape within tumors is much more diverse and complex than originally thought [22]. Based on the expression pattern of certain genes (chemokines, chemokine receptors, and lineage-specific molecules), they identified many different populations exerting both inflammatory and anti-inflammatory functions [22]. Chevrier et al. also demonstrated, by single-cell transcriptomics, that within the myeloid population in renal cell carcinoma (RCC), the resident myeloid cells like macrophages display both anti-tumor and pro-tumor markers [23]. Both teams also proved that the tissue-resident myeloid populations differ in the expression pattern of certain markers in comparison with their peripheral blood counterparts [22,23]. These results are indicative of the complexity and diversity of the myeloid cell populations present in both the periphery and in the afflicted tissue, proving that phenotypic characterization of TIM cells and MDSCs is a rather difficult task.

## 2. Development of MDSCs

It is considered a common notion that chronic inflammation promotes tumor development through various mechanisms. These mechanisms may include pro-angiogenic factors, matrix metalloproteinases (MMPs), damaged associated molecular pattern molecules (DAMPs), and activation of signaling pathways activated by a different combination of molecules existing in the peripheral blood and the TME [24].

The TME network bares certain characteristics that, altogether, facilitate the development of cancer cells, as well as the induction and expansion of MDSCs. To begin with, the TME is as highly hypoxic environment where MDSCs can easily thrive. It has been demonstrated that MDSCs upregulate hypoxia-induced factor 1α (HIF1α), which in turn is translocated in the nucleus, binding to the hypoxia response element upstream of certain genes crucial for cancer progression [2,24]. Some of these targets are vascular endothelial growth factor (VEGF), inducible nitric oxide synthase (iNOS), MMPs, and arginase 1 (ARG1). The observed overexpression of HIF1α by MDSCs enables them to perform their immunosuppressive ability in the TME [24]. Moreover, MDSCs express certain receptors for growth factors. The activation of these receptors is responsible for the development and function of MDSCs. The main pathway through which they are developed is the Janus kinase (JAK)/Signal Transducer and Activator of Transcription (STAT) pathway, with the members of the STAT protein family being the main downstream signal transductors activating transcription factors such as nuclear factor kappa-light-chain-enhancer of activated B cells (NF-κB), interferon regulatory factor-8 (IRF-8), CCAAT-enhancer-binding proteins (C/EBPβ), and hypoxia-inducible factor 1-alpha (HIF-1α) [2,15,24,25]. The above signaling cascade can be activated by various factors situated in the TME, with the most common among them being growth factors secreted by cancer cells, pro-inflammatory cytokines, calcium binding proteins (calgranulin A/B; S100A8/A9), heat shock protein (HSP72), high-mobility group box 1 (HMGB1), and other molecules in the TME [15,24,25].

### 2.1. VEGF-Driven MDSC Development

A most distinguishing example of a receptor involved in MDSC development is the VEGF receptor (VEGF2R). VEGF is a growth factor secreted by cancer cells and which enables neo-angiogenesis and metastasis. The circulating VEGF emitted in the TME acts as a chemo attractant for MDSCs [5,18,26]. Specifically, VEGF attracts the MDSCs to migrate from the BM to the periphery, thus increasing their presence in blood circulation [27]. Binding of the VEGF to its receptor is associated with increased production of reactive oxygen species (ROS) via the activation of the JAK2/STAT3 pathway. MDSCs themselves can produce VEGF, creating a positive autocrine feedback loop [24].

### 2.2. G-CSF and GM-CSF-Driven MDSC Development

Other factors responsible for the development of MDSC are the granulocyte colony-stimulating factor (G-CSF) and the granulocyte–monocyte colony-stimulating factor (GM-CSF) [5,26]. G-CSF binds to the CSF3 receptor (CSF3R), activating a JAK/STAT downstream signal transduction. This signal activates the transcription factors MyC and C/EBP that promote the development and immunosuppressive functions of PMN-MDSC [28]. Similar to G-CSF, GM-CSF has its own receptor, the GM-CSF2 receptor (CSF2R). Binding of its ligand to the CSF2R activates the JAK/STAT pathway, and high concentration of GM-CSF is correlated with generation and mobilization of M-MDSC and PMN-MDSC from the bone marrow and increased immune suppression [24,29].

### 2.3. Cytokine-Driven MDSC Development

Cytokines can facilitate the recruitment and activation of MDSCs in the TME, such as transformation growth factor β (TGFβ), interleukin-(IL)-4, IL-13, IL-28 (IFN-λ), IL-17, IL-10, and IL-1β [17,24,30]. As it seems, by means of experiments conducted on tumor-bearing mice, IL-1β is a potent inducer of MDSCs [24]. Not only does IL-1β aid in the accumulation of MDSCs, but it also commences the production of other molecules necessary for the expansion of MDSCs (e.g., VEGF, IL-6, GM-CSF) [24]. In the TME, there is an abundance of TGF-β, which is able to generate MDSC populations, as well as prime other myeloid lineages to more suppressive ones [5,31]. Afterwards, MDSCs themselves are able to produce TGF-β, creating a feedback loop that sustains their antitumor immunity [31]. S100A8/A9 is a pro-inflammatory stimulator that activates the STAT3/5 pathway responsible for keeping the immature myeloid cells from differentiating into their mature lineages, which makes it also responsible for activating MDSC’s suppressive mechanisms [24,25].

## 3. Accumulation of MDSCs to Tumor Sites

Tumor niches demonstrate an abundance of different cytokines and chemokines that are implicated in the recruitment of immunosuppressive cells. C–C motif chemokine ligand 2 (CCL2) was initially characterized as a cytokine, which, upon interaction with its correspondent receptor, CCR2, on circulating monocytes, could expedite chemotaxis to areas of inflammation [21,32]. It has been shown that in in vitro experiments of human cancer models, secretion of tumor-derived CCL2 attracts CCR2 expressing MDSCs towards the cytokine [21], with evidence provided by Guan et al. positively correlating the amount of CCL2 to MDSC accumulation and immunosuppressive capacity [33]. In a similar way, the chemokine interleukin-8 (IL-8, CXCL8) is a chemo attractant that was found to be released by cancer cells and to ulcerate cells of myeloid lineages upon binding to G protein-coupled receptors C–X–C motif chemokine receptor 1 and 2 (CXCR1 and CXCR2) [32,34], while the CCL3/CCR5 axis was shown to aid in the maintenance of immunosuppressive myeloid cells to the tumor niches [21]. Guan et al. also highlighted that IL-17, a pro-inflammatory cytokine mainly secreted by Th17 cells, is over expressed by malignant cells and promotes the translocation of MDSCs from the periphery to the tumor site [33].

In addition, the highly hypoxic TME contributes to the accumulation of MDSCs. In a more specific manner of speaking, HIF-1α was shown to induce the expression of ectonucleoside triphosphate diphosphohydrolase 2 (ENTPD2), commonly known as CD39L1, aiding their movement towards the TME [35]. In the hypoxic TME, VEGF is thought to be the dominant chemoattractant for MDSCs, as it was shown that, both in mice and in non-small cell lung cancer (NSCLC) patients, hypoxia upregulates its expression and aids in MDSC accumulation [36]. This process of VEGF-mediated attraction to the TME is possible by means of VEGFR expression on MDSCs [37]. Primary tumor clusters can potentially gather MDSCs from the BM by releasing exosomes. The exosomal content is able to reprogram the target cell, leading to increased mobility of the progenitor myeloid populations to the tumor site [18,38].

## 4. Immunosuppressive Functions of MDSCs

MDSCs are active mediators of the immune system, executing their various function under different circumstances. As it happens, cancer cells exploit MDSCs in order to escape the surveillance of the immune system and, thus, maintain their presence in the tissue [2,5,39,40]. Specifically, MDSCs are recruited by tumor cells in order to pave the way for the expansion of the first. This action is mediated by distinct mechanisms such as the secretion of inhibitory and anti-inflammatory cytokines (e.g., IL-10, TGF-β, IL-6, IL-28) and of ROS, expression of iNOS (also known as NOS2) and arginase 1 (ARG1), along with collaboration with other cells with repressive activity like T regulatory cells (Tregs) and Th17, and the expression of immune checkpoint inhibitors (Table 1) [3,5,6,41,42,43].

### 4.1. ROS, iNOS, and ARG-1

The above means of suppressive actions is exerted by either M-MDSCs or PMN-MDSCs, or, in some cases, by both of them. It was demonstrated that ROS are mainly produced by PMN-MDSCs [14]. The sources of intracellular ROS, in an MDSC, are mitochondria and NADPH oxidase, the peroxisome, and various metabolic procedures [44]. ROS are under the auspices of the STAT3 transcription factor and are associated with the metabolism of L-arginine, but mostly with the NADPH oxidase superfamily [2,5,42]. In the TME, the STAT3/ROS signaling pathway is activated by growth factors secreted by cancer cells. S100A8/A9 stimulates the production of ROS in a STAT3-dependent manner, leading to nitration of the TCRαβ, thus rendering the T cells incapable of interacting with the antigen bound in the major histocompatibility complex II (MHC-II) of the antigen-presenting cells (APCs) and initiating the anti-cancer response [24]. The produced ROS hamper the function of CD8 molecules and downregulate the expression of CD3ζ-chain [44]. The above mechanism results in the inability of T cells to become activated [24,44], while the oxidative stress leads the target cell to apoptosis [36]. Finally, elevated levels of ROS are associated with the expression of VEGF receptor, and thus, their recruitment to the TME [35].

Another mechanism responsible for oxidative stress in the TME is the iNOS enzyme; M-MDSCs are mainly responsible for the expression of iNOS [14]. Similar to ROS, the production of iNOS is controlled by signaling pathways mediated by Th1 cytokines (IFN-γ, IL-1β), as well as the hypoxia existing in the TME [2,45]. In order for iNOS to act, NADPH oxidase must be present. Synergistically, by metabolizing l-arginine (l-Arg), they produce reactive nitrogen species (RNS) like NO. It was shown that in conditions were L-Arg is in low concentration, MDSCs promote the production of peroxynitrite via iNOS [35]. In a similar way, like the production of ROS, S100A8/A9 through activation of STAT1 facilitates the production of iNOS [24]. However, ROS and NO cannot coexist and, thus, it is turned into ONOO-, which hampers the TCR function of T cells by nitration [2,24]. The highly reactive RNS can drive T cells to apoptosis [35].

Additionally, MDSCs have the ability to reduce the levels of certain amino acids in the TME (e.g., l-Arg, l-Trp, and l-Cys) [24]. This immunosuppressive mechanism exists in the concept of MDSC-induced amino acid depletion in the TME [2,24,46]. The ARG1-mediated depletion of l-Arg from the TME hinders the ability of the T cell population to exert their antitumor functions, since l-Arg is critical for the production of the CD3ζ-chain, with the latter being a crucial component for a functional TCR [24]. This enzyme, the expression of which is under the control of PGE2 and Th2 cytokines (IL-4, IL-10, IL-13), metabolizes l-Arg into l-ornithin and urea [2,24,35]. Apart from amino acid depletion, ARG1 also exerts an immunosuppressive ability by establishing a state of oxidative stress. The result of l-Arg metabolism from ARG1 is the production of NO, ROS, and RNS, all of these being factors that suppress the T cell population, thus rendering them unable to initiate an anti-cancer response [46]. Along with the ARG1, MDSCs translocated to TME due to inflammation also express the cationic amino acid transporter 2 (CAT2), further depleting the available l-Arg [46].

### 4.2. Anti-Inflammatory Cytokine Production, Exosomes, and Immune Checkpoint Regulation

MDSCs promote immunosuppression by secreting anti-inflammatory cytokines [21]. IL-10, which is produced by the MDSCs, arrests the production of interferon-γ (IFN-γ) by CD4^+^ T cells, along with promoting the metastasis of cancer cells [2,19,24]. The production of IL-10 is further upregulated by HMGB1 [24,47]. MDSCs have the capacity to produce TGF-β [48], either as secreted or as membrane-bound protein, by means of which they render natural killer (NK) cells inactive, whilst facilitating the accumulation and induction of Tregs, which further induce the effector T cell suppression against the progression of cancer [2,6,24,49]. Some researchers also demonstrated the interaction between CD8/Tc17 and CD4/Th17 cells, with IL-17R expressing MDSCs [50]. This interaction enables MDSCs to accumulate in tumor sites, and further induces neutrophils to acquire an MDSC-like immunosuppressive phenotype to inhibit the infiltration of CD8^+^ T cells in the TME [50]. The latter process is mediated by the upregulation of iNOS in MDSCs, TAMs, and MDSC-like neutrophils [51,52]. IL-17 was also shown to promote the expression of effector genes in MDSC, such as MMPs, VEGF, which promote angiogenesis [52,53]. MDSCs were shown to produce IL-28 [54], while IL-13 production from M-MDSCs in tumor-bearing mice is able to prevent the antigen presentation from CD8^+^ T cells [5,30].

Another modus operandi that MDSCs seem to exploit in order to exert immunosuppression is the release of exosomes. The exosomal cargo of MDSCs is rather rich in multiple molecules (e.g., cytokines and growth factors), which exert certain suppressive and tumor-favoring functions [25,36,49]. Apart from some anti-inflammatory mediators (TGF-β, IL-10, S100A8/A9, HMGB1, MMPs), they also contain small RNA molecules known as micro RNAs (miRNAs) that either induce other MDSCs to exert immunosuppressive functions (e.g., miR-210, responsible for the expression of ARG1) or aid in the process of angiogenesis (e.g., miR-126a) [25,36,49]. Recent studies have demonstrated that MDSCs express immune checkpoint regulators, and especially programmed-death ligand 1 (PD-L1), which binds to programmed death 1 receptor (PD-1) [1,2,55,56]. PD-1 is expressed by immune effector cells like CD4^+^ T cells and CD8^+^ T cells, antigen-presenting cells (APCs) and dendritic cells (DCs), both in the periphery and in the tumor site [55]. PD-L1^+^ MDSCs interact with the PD-1^+^ T cells, rendering them inactive in a senescent-like state, where the cytokine production is arrested. This interaction between PD-1 and PD-L1 initiates the apoptosis sequence in lymphocyte lineages [1,2,6,42]. In the TME, however, this case is quite different. In contrast with the periphery, TME is highly hypoxic. Under these circumstances, PD-L1 is overexpressed by MDSCs, since the PD-L1 gene is under the control of HIF-1α [2]. Antonios et al. demonstrated that tumor-infiltrating myeloid cells (TIMs) were able to hamper the cytolysis mediated by tumor infiltrating lymphocytes (TIL) in a PD-1/PD-L1-dependent manner [56].

## 5. MDSC-Induced Angiogenesis

**Figure 1:**
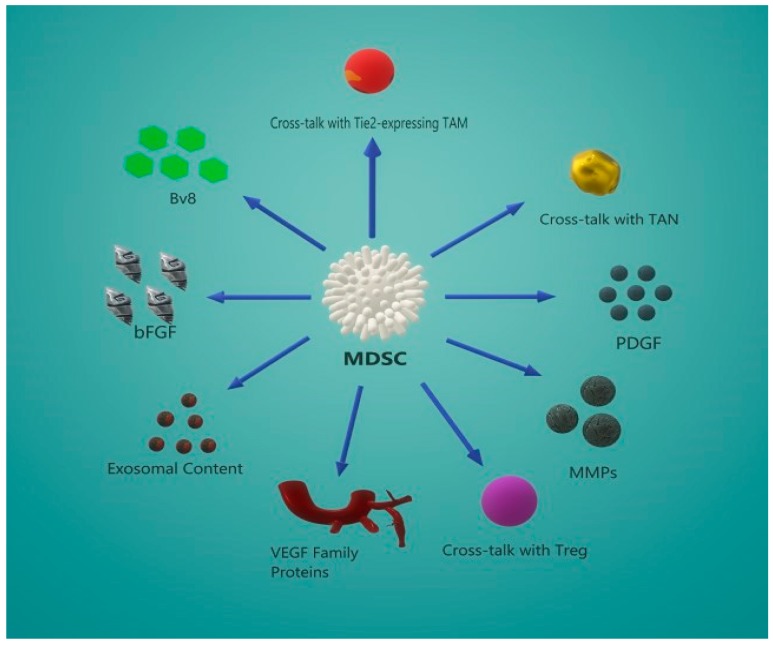
MDSCs promote de novo angiogenesis via different mechanisms. Mainly, MDSCs promote neo-angiogenesis by secreting growth factors like VEGF, bFGF, Bv8, and PDGF. Additionally, they remodel the extracellular environment via MMP production. Moreover, they are capable of reprogramming other cells to a tumor-promoting phenotype (Tie2^+^TAM, TAN, and Treg), which in turn can promote angiogenesis via the secretion of proangiogenic factors like VEGF. Recently, MDSCs were found to promote vascularization by means of exosome. The exosomal content is vast, and some of the molecules it contains can either prime target cells to acquire a proangiogenic phenotype, or induce angiogenesis, since they also contain proangiogenic factors (VEGF-A, miRNA-126α).

In order for a tumor cell to disseminate and re-establish a new colony, first and foremost, it needs to build a network of vessels from the original tumor niche to the blood circulation to facilitate its journey throughout the human body. Angiogenesis is the process of creation and maintenance of the vasculature during development and in malignant conditions like cancer. The process of extravasation is a complex one and, apart from the tumor cells, it also includes immunosuppressive cells. MDSCs were proven to subvene the process of de novo vascularization by a plethora of different, and sometime overlapping, mechanisms (Figure 1) [18,57].

### 5.1. The VEGF/VEGFR Angiogenic Pathway

The most common and well-studied mechanism of MDSC-mediated angiogenesis is the production of VEGF [36]. The VEGF family of proteins consists of VEGF-A, -B, and -C [37,58]. MDSCs exploit the abundance of VEGF in the TME, and via the tyrosine kinase receptor VEGFR, they are able to initiate a signaling cascade implicating JAK2/STAT3, resulting in the production of even more angiogenic molecules [59,60]. VEGF-A most commonly binds to VEGFR1 and VEGFR2 [61]. VEGFR2 can initiate a far more potent signal compared to VEGFR1, even though the latter has a stronger affinity for VEGF-A [61]. MDSCs were shown to express both VEGFR1 and VEGFR2 in an ovarian cancer mouse model, but intriguingly, tumor tissue-resident MDSCs were shown to strongly express VEGFR2 [62]. Interestingly, VEGF-activated MDSCs exceed a more potent immunosuppressive activity in relation to ones not exposed to VEGF [63].

As mentioned above, tumor-derived factors (VEGF, IL-6, IL-10) stimulate and recruit MDSCs to the TME. In turn, MDSCs via a STAT3-mediated pathway can generate more VEGF, establishing a positive feedback loop, which sustains their population and their angiogenic activity [18,63,64]. Casein kinase 2 (CK2) production, which is considered to be an angiogenic contributor, is also under the auspices of STAT3, both in MDSCs and cancer cells [65]. C/EBP-δ plays a key role in VEGFR2-mediated angiogenesis. It is a protein critical for myeloid cell development, and in tumors, MDSCs overexpress it, which leads to the increased expression of VEGFR2 from the adjacent endothelial cells [66]. This could be an indirect angiogenic MDSC-promoted mechanism.

### 5.2. Secondary Angiogenic Mechanisms

The VEGF/VEGFR axis is not the sole angiogenic mechanism in the MDSC arsenal. After VEGF signaling activation, MDSCs begin to generate pro-angiogenic proteins known as MMPs, a rather big family of proteins, including MMP2, MMP8, MMP9, MMP13, and MMP14 [18,59,63,67]. MMPs are extracellular matrix remodeling enzymes that, once released from the cell, begin to digest the extracellular environment, facilitating the extravasation process [57]. MMP9 is considered to be the master regulator among the metalloproteinases. It is produced as an immature pro-angiogenic molecule by MDSCs, in collaboration with TAMs and TANs [57,68,69]. miRNA-494 was also shown to increase tumor invasiveness by MDSC-derived MMP overexpression [68]. In fact, Heusschen et al. demonstrated that angiogenesis was mainly performed by the MMP9-producing PMN-MDSC population [67].

Some other factors contributing to MDSC-related angiogenesis and pre-metastatic enablement are bombina variegata peptide 8 (Bv8) [18,58,63], platelet-derived growth factor (PDGF) [58,59] and basic fibroblast growth factor (bFGF) [58,63]. These factors are hailed as alternative vasculature-creating mechanisms, but equally effective and perhaps overlapping, since they use similar signaling pathways (extracellular-related kinase (ERK)/Akt or mitogen-activated protein kinase (MAPK)), while the tumor-activated pSTAT3 pathway in MDSCs results in their generation [58,64]. Kuo et al. detected that CXCL17 supported de novo angiogenesis and lung metastasis by means of PDGF expressing CD11b^+^Gr-1^+^ MDSC in mice [70].

The IL-1 family of cytokines (previously hailed as hemopoietin-1) is also implicated in extravasation [71]. Both homologues, IL-1α and IL-1β, were reported to demonstrate angiogenic activities, with IL-1β being able to induce the expression of VEGF [71]. IL-1β further induces the expression of both VEGF and VEGFR in a HIF-1α-dependent way, even under normoxia, whereas IL-1α derived from gastric and colonic tumor cells could trigger de novo in vitro [71]. Pingwara et al. demonstrated that IFN-λ2 expression was increased in MDSCs isolated from the lungs of tumor-bearing mice and stimulated tube formation in an in vitro assay [72]. In the same research, they also found that IFN-λ2 was increased in MDSCs purified from peripheral blood of advanced cancer patients, suggesting that MDSCs promote tumor progression and angiogenesis in an IFN-λ2-dependent signaling [72].

### 5.3. Crosstalk Between MDSCs and Other Effector Cells

The general crosstalk between MDSCs (either by secretion of soluble factors or by exosomes) and cancer cells results into the production of pro-angiogenic factors (VEGF, MMP, miRNA-126α), preparing the way for cancer cells to metastasize [3,5,6,49]. The interaction of IL-28 with tumor cells can promote the epithelial to mesenchymal transition (EMT) of the cancer cells, making the latter more invasive, since it also upregulates the expression of VEGF by tumor cells in canine mammary cancer cells [54]. MDSCs are extremely interactive cells and, as such, they can also prime other terminally differentiated cells into a tumor-promoting phenotype.

TAMs and TANs constitute a characteristic example of such a switch. They were originally circulating cells in the blood stream, but after reacting upon pro-tumorigenic factors secreted from both cancer and immunosuppressive cells alike, they acquired a tumor-favoring phenotype [1,69,73]. During this turnover, M2 and N2 macrophages and neutrophils, respectively, produce pro-angiogenic factors such as VEGF and MMPs [69,73,74,75,76]. Furthermore, Deryugina et al. proved that TANs produce more MMP9 compared to TAMs, indicating that TANs might play a crucial role in metastasis [73]. The Tie2-expressing TAM population seems to be most interactive to MDSCs crosstalk. Tie2^+^ ΤAMs are recruited by angiopoietin 2 (Ang-2), further fostering an angiogenic microenvironment [57]. TAMs are also capable of promoting angiogenesis in a Notch/IL-1β-dependent manner, with Notch signaling initiating the expression of both VEGF and VEGFR [77].

An additional interactive network is formed between MDSCs and Tregs [24,35]. Consequently, Tregs are accumulated in the TME and produce VEGF, enriching the pro-angiogenic pool [35,60]. Finally, a cross-talk between MDSCs and mast cells was also shown to promote angiogenesis. In gastric cancer conditions, mast cells were reported to realize tumorigenic-promoting functions like extravasation and lymphagiogenesis, in addition to recruiting more MDSCs to the TME [78].

### 5.4. MDSC-Derived Exosome Content Promotes Angiogenesis

Last but not least, exosomes have risen as a novel immunosuppressive and tumor-promoting mechanism. MDSCs exert some of their immunosuppressive and tumor-promoting functions by secreting vesicles rich in effector proteins [25,49,79]. The cargo of these nanovesicles may differ, thus alternative combinations of proteins and miRNAs may be distributed unequally to the exosome that are to be released from the plasma membrane [25,79]. VEGF, TGF-β, and S100A8/A9 were shown to be enriched in the exosomes derived from MDSCs, and were able to polarize M1 macrophages to tumor-promoting M2 phenotype and expedite invasion and metastasis [25,79,80].

The RNA content of MDSC-derived exosomes was also dimmed as an angiogenetic contributor. A combination of miRNAs and long non-coding RNAs (lncRNAs) that exist in the exosomes exert pro-angiogenic function by reprogramming the target cell [25,38]. Most notably, miRNA-126α, from MDSC-derived exosomes and tumor-derived exosomes (TEXs), was able to sustain MDSCs and promote angiogenesis, while conveying chemoresistance [25,38,80,81]. Specifically, miR-155 and miR-21 induced MDSCs in the TME to acquire a more immunosuppressive function, while simultaneously facilitated tumor growth via activation of the STAT3 pathway [25,64]. miR-9 contained in MDSC-derived exosomes and TEXs was shown to increase the immunosuppressive activity of MDSC and promote angiogenesis by reprogramming endothelial cells [49,81]. Moreover, the HOX transcript antisense intergenic RNA (HOTAIR) was also associated with tumor growth and angiogenesis, as well as for the CCL2-mediated recruitment of MDSCs and TIMs (namely, TAMs) [82]. Mast cells were also shown to increase prostate tumor cell invasion via upregulating MMP expression in a HOTAIR-mediated manner [83]. Still, little is known regarding the role of lncRNAs and their effect in tumor progression and angiogenesis.

## 6. MDSC Targeting for Cancer Therapy

Understanding the role and functions of MDSCs in cancer-related conditions is of vital importance. Every day, more and more therapeutic approaches are designed targeting MDSCs in an attempt to exterminate cancer cells [32]. These approaches are immunotherapy, chemotherapy, or radiation therapy, or it could be a combination of the above [84]. Some of these approaches are currently undergoing clinical trials to estimate the efficacy and the safety of their application in cancer patients (Table 2). These trials involve both direct and indirect MDSC depletion and blood vessel shrinkage. Blocking of several mechanisms that are used by MDSCs to treat cancer were examined by various trials.

Other approaches include the maturation of MDSCs to their terminal lineages (macrophages, DCs, and neutrophils), blockade of VEGF signaling, and combinational treatments [84,85]. Treatment with vitamin E reduced the NO-mediated immunosuppressive function of MDSCs and enhanced NK cytotoxicity [63]. Consequently, treatment with all-trans-retinoic acid (ATRA) induces the differentiation of MDSCs to DCs and macrophages reducing their immunosuppressive activity, while ameliorates the efficacy of anti-VEGFR2 immunotherapy [63,86]. Bennewith’s group demonstrated the importance of monitoring the presence of MDSCs in a mice model of metastatic breast cancer, indicating the role of MDSCs in correlation with increased metastatic potential [87]. It constitutes a common notion that multiple signaling pathways are involved in cancer metastasis and angiogenesis. The JAK2/STAT3 is one that has drawn many researchers’ attention, since it mediates angiogenesis in both cancer cells and MDSCs [65]. Since the immunosuppressive and angiogenic functions of MDSCs are regulated by the JAK2/STAT3 signaling cascade, it is only rational to assume that its inhibition could rescue immunosuppression and extravasation.

In the light of the realization of the role of MDSCs in cancer progression, many studies have tried to unmask different approaches. Knowing that MDSCs promote vascularization, treatment options targeting angiogenic pathways are imperative. In fact, many studies have demonstrated the efficacy of such procedures. Previous work from our group demonstrated that anti-VEGF-based combinational chemotherapy had an impact on PMN-MDSC reduction in the peripheral blood of NSCLC patients [27]. Ko et al. also demonstrated the reductive effect of sunitinib, a receptor tyrosine kinase inhibitor, on MDSCs in renal cell carcinoma patients [85]. Consequently, Liu and co-workers investigated the effect of JAK2/STAT3 blockade on MDSCs. They discovered that inhibition of this signaling cascade in head and neck squamous cell carcinoma (HNSCC)-bearing mice minimized the production of VEGF and CK2, subsequently leading to the reduction of vessel formation [65]. Moreover, Bauer et al. showed that treatment of MDSCs with all-trans retinoid acid (ATRA) hampered their accumulation and decreased the production of MMP9, indicating the possible effect of ATRA in vessel normalization and MDSC reduction [88]. Other experimental approaches targeting MDSCs in the concept of cancer include the blockade of accumulation pathways like the CCL/CCR2 axis and IL-8/CXCR1/2 interactions, which may hinder the trafficking of MDSCs to the TME and inhibit extravasation [32]. Overall, current studies regarding anti-VEGF treatment are centered on the production of tyrosine kinase inhibitors in order to arrest the activation of VEGFR-mediated vascularization and other secondary angiogenic pathways using the same signaling transducers [89].

Although many clinical trials have been conducted in the hope of curing cancer, not all of them are crowned winners. Nonetheless, a research group demonstrated in a clinical trial (NCT02922764) (Table 1) that depletion of MDSCs leads to cancer reduction. Tavazoie et al. researched the impact of liver-X nuclear receptor (LXR)/Apolipoprotein E (ApoE) signal on MDSCs, and what they found was astonishing. Treatment of MDSCs with agonist of LXR/ApoE, led to the depletion of MDSCs in in vitro as well as in in vivo models. Namely, the RGX-104 LXR agonist reduced the amount of PMN-MDSCs and M-MDSCs in the tumor site and in the periphery by initiating apoptosis, whereas in ApoE-deficient mice, there was no observable MDSC depletion, indicating that ApoE is necessary for the killing of MDSCs in cooperation with LXR agonism. Thus, Tavazoie et al. suggested that synergistically, LXR/ApoE are efficient in targeting and eliminating MDSCs in the context of cancer [90].

However, taking into consideration that in the TME, MDSCs are the only tumor-promoting populations, many studies have focused on targeting TAMs and TANs. Indeed, TAMs are able to induce extravasation in tumor sites, thus targeting them is imperative to fight tumor growth and metastasis [91]. In this concept, many approaches have been tested, including the common TIKs that are widely used, but the results are not that promising [91]. Nonetheless, Penn et al. demonstrated that blockade of VEGF-C, which is secreted by TAMs, limited lymphagiogenesis and depleted TAMs [92]. As far as TANs are concerned, it was shown that they exert an anti-VEGF function. Specifically, Seeger et al. showed that the presence of CD177^+^ neutrophils in colorectal cancer patients is associated with poor prognosis [93]. Finally, blocking of the Ang2/Tie axis was shown to reduce the tumor-promoting functions and recruitment of TAMs [92,94], and could be used as credentials denoting that blocking of secondary angiogenic mechanisms could improve anti-VEGF treatment [93].

## 7. Conclusions and Future Perspectives

In conclusion, the role of MDSCs in cancer firstly lies in the establishment of an immunosuppressive environment, and secondly, in the remodeling of the TME in order to initiate tumor invasiveness and metastasis. During cancer progression, MDSC-mediated extravasation is exerted by many different pathways, but first and foremost, by the production of VEGF and MMPs. Nonetheless, secondary mechanisms also exist to support the angiogenic process, resulting in the composition of a complex signaling network. Considering that the MDSC population is vast and exerts its functions in different manners, it is relatively difficult to devise a therapeutic approach to diminish their numbers or arrest them in the peripheral blood and in the TME. Regardless, many studies have demonstrated the efficacy of MDSC targeting and the possibility of overcoming the boundaries in MDSC-reducing cancer treatment. Further studies are in order to reinforce the arsenal of knowledge about MDSCs and to better understand the possibilities of combinational treatments in response to MDSC-supported cancer progression.

## Figures and Tables

**Table 1:** Immunosuppressive mechanisms of myeloid-derived suppressor cells (MDSCs).

Mechanism	Mediated By	Effect
Induction of immunosuppressive cells	Release of IFN-γ, IL-10, and TGF-β	Induction of Tregs
	Release of IL-10	Generation of M2 macrophages
Impaired lymphocyte homing	Cleavage of L-selectin by the metalloprotease ADAM 17	Reduction in the homing and antigen-dependent activation of CD8^+^ T cells in lymph nodes
	Downregulation of CD44 and P-selectin by NO-producing M-MDSC	Blocking of T cell extravasation and tissue infiltration
Production of reactive oxygen species (ROS)	NADPH oxidase 2 (NOX-2)	Reduced CD3ζ-chain expression Inhibition of T cell proliferation Increase of ARG1 expression
Nitric oxide production	Induction of COX-2 expression Induction of HIF-1α expression Increase of ARG1 expression	Induction of T cell anergy
	Induction of nitrogen species	Induction of T cell apoptosis TCR nitration Chemokine nitration
Cysteine/cystine and L-arginine deprivation	Increased uptake of L-arginine by the CAT2B transporter	Reduced TCR ζ-chain expression Inhibition of T cell proliferation Increase of ARG1
	Increased uptake of cysteine via SLC7A11 transporter	Reduced protein synthesis Glutathione production
Adenosine production	Induction of the ectoenzymes CD39 and CD73 via TGFβ and hypoxia	Decreased phosphorylation of Zap70, ERK, and Akt Reduced expression of CD95L, perforin, IFN-γ, TNF-α, CD25 in T cells
Activation of immuno-regulatory molecules	High expression of B7	T cell anergy
	High expression of PD-L1	T cell apoptosis
	High expression of FasL	Upregulation of Fas receptor

**Table 2:** Clinical trials involved in MDSC depletion and modulation in cancer.

Title	Malignancy	Treatment	Trial No	Phase
A Study of RGX-104 in Patients with Advanced Solid Malignancies and Lymphoma	Malignant neoplasms	RGX-104; Nivolumab Ipilimumab; Docetaxel Pembrolizumab Carboplatin Pemetrexed	NCT02922764	Phase I
Trial to Evaluate Safety and Efficacy of Vinorelbine with Metronomic Administration in Combination with Atezolizumab as Second-line Treatment for Patients with Stage IV Non-small Cell Lung Cancer (VinMetAtezo)	Non-small cell lung cancer	Atezolizumab Vinorelbine	NCT03801304	Phase II
Dendritic Cell Vaccine with or Without Gemcitabine Pre-Treatment for Adults and Children with Sarcoma	Sarcoma	Dendritic Cells Vaccine	NCT01803152	Phase I
	Soft tissue sarcoma	Lysate of Tumor		
	Bone sarcoma	Gemcitabine Imiquimod		
Capecitabine + Bevacizumab in Patients with Recurrent Glioblastoma	Glioblastoma	Capecitabine Bevacizumab	NCT02669173	Phase I
